# Quantification of damage in DNA recovered from highly degraded samples – a case study on DNA in faeces

**DOI:** 10.1186/1742-9994-3-11

**Published:** 2006-08-16

**Authors:** Bruce E Deagle, J Paige Eveson, Simon N Jarman

**Affiliations:** 1 School of Zoology, University of Tasmania, Box 252-05, Hobart, Tasmania, Australia; 2 Australian Antarctic Division, Channel Highway, Kingston, Tasmania, Australia; 3 CSIRO Marine and Atmospheric Research, Box 1538, Hobart, Tasmania, Australia

## Abstract

**Background:**

Poorly preserved biological tissues have become an important source of DNA for a wide range of zoological studies. Measuring the quality of DNA obtained from these samples is often desired; however, there are no widely used techniques available for quantifying damage in highly degraded DNA samples. We present a general method that can be used to determine the frequency of polymerase blocking DNA damage in specific gene-regions in such samples. The approach uses quantitative PCR to measure the amount of DNA present at several fragment sizes within a sample. According to a model of random degradation the amount of available template will decline exponentially with increasing fragment size in damaged samples, and the frequency of DNA damage (λ) can be estimated by determining the rate of decline.

**Results:**

The method is illustrated through the analysis of DNA extracted from sea lion faecal samples. Faeces contain a complex mixture of DNA from several sources and different components are expected to be differentially degraded. We estimated the frequency of DNA damage in both predator and prey DNA within individual faecal samples. The distribution of fragment lengths for each target fit well with the assumption of a random degradation process and, in keeping with our expectations, the estimated frequency of damage was always less in predator DNA than in prey DNA within the same sample (mean λ_predator _= 0.0106 per nucleotide; mean λ_prey _= 0.0176 per nucleotide). This study is the first to explicitly define the amount of template damage in any DNA extracted from faeces and the first to quantify the amount of predator and prey DNA present within individual faecal samples.

**Conclusion:**

We present an approach for characterizing mixed, highly degraded PCR templates such as those often encountered in ecological studies using non-invasive samples as a source of DNA, wildlife forensics investigations and ancient DNA research. This method will allow researchers to measure template quality in order to evaluate alternate sources of DNA, different methods of sample preservation and different DNA extraction protocols. The technique could also be applied to study the process of DNA decay.

## Background

An increasing number of zoological studies use DNA derived from poorly preserved, decomposed or ancient tissue sources – examples include ecological studies using genetic material from faecal samples [e.g. [1,2]], wildlife forensic investigations examining processed animal products [e.g. [3]], and evolutionary studies using DNA from historic museum skin collections [e.g. [4]] or fossilized bones [e.g. [5]]. Often only small amounts of DNA can be extracted from such samples and it is invariably highly damaged. In the absence of normal cellular processes, DNA strand breakage rapidly begins to occur as a result of endogenous endonuclease activity and spontaneous depurination [6]. Depending on the ambient conditions further strand breaks, oxidative damage and molecular crosslinks accumulate [7-9]. Assessing the extent of damage is difficult, especially when the DNA of interest is present in a sample containing DNA from several different sources. However, determining DNA quality is desirable in many situations, as reflected by the variety of approaches that have been used to measure DNA damage [4,7,9-15].

Qualitative estimates of DNA fragment sizes can be obtained through gel electrophoresis followed by visualisation of fragments [e.g. [7,12]]. This approach is simple but has limited sensitivity and, because it does not differentiate between fractions of the DNA extractions, it is generally only useful if all DNA present has been equally degraded. Another approach commonly used to assess DNA quality is through observations of the decrease in PCR amplification signal from PCR targets of increasing sizes [e.g. [4,16,17]]. Since double-strand breaks and many other forms of DNA damage block the extension step of PCR [8,14], the ability to recover large fragments via PCR indicates relatively low levels of DNA damage. By determining the maximum amplifiable fragment size in different samples it is possible to compare relative amounts of DNA degradation. There are several related PCR-based methods used to measure DNA damage incurred by exposure to mutagenic compounds [10,18-20]. These techniques, often called PCR-stop assays, measure gene-specific damage by quantifying the decrease in the number of molecules that can be amplified following a particular genotoxic treatment. A limitation of the currently used PCR-stop assays is that the total amount of target DNA has to be quantified using PCR independent means, or else a dose-response curve needs to be constructed. This precludes their use in a number of situations, such as when the DNA of interest is present at low concentrations or in a mixture with non-target DNA.

**Figure 1:**
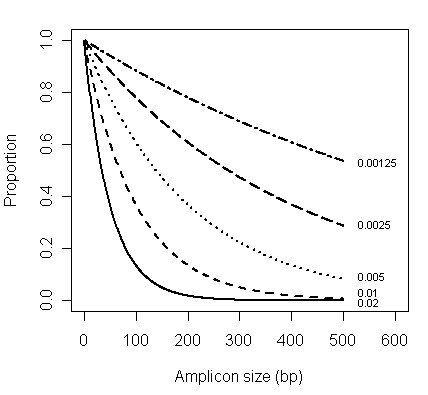
**Theoretical proportion of amplifiable fragments versus amplicon size after a random degradation process**. Results are shown for cases in which the probability of a nucleotide being damaged (λ) is: 0.00125, 0.0025, 0.005, 0.01 or 0.02.

**Figure 2:**
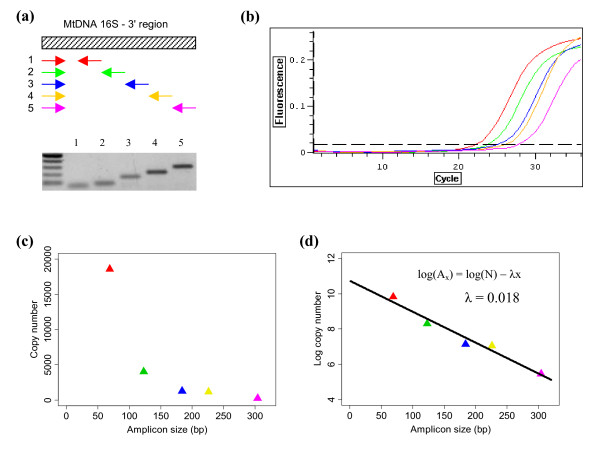
**Overview of the approach for quantification of DNA damage**. (a) Primers are designed that amplify fragments of several sizes. The schematic representation shows the position of oligonucleotides and the picture below shows the corresponding PCR products (amplified from genomic DNA) separated on a 1.8% agarose gel. (b) The various sized fragments are amplified using real-time PCR. This representative plot of fluorescence observations shows amplification of herring DNA from a single sea lion faecal DNA extraction. PCR fragment sizes from left to right are 69 bp, 123 bp, 184 bp, 226 bp and 304 bp. (c) Copy number (Ax) estimates are obtained for each of the amplicon sizes (x). The plot shows the amount of herring DNA in a sea lion faecal sample (#7). (d) The data is then log-transformed and a linear model is fitted in order to estimate the probability of a nucleotide being damaged (λ).

Here, we extend the existing PCR-based methods by proposing an experimental strategy that can be used to quantify gene-specific DNA damage in dilute, mixed template samples. The approach uses real-time quantitative PCR (qPCR) to measure the amount of amplifiable target DNA for fragments of various sizes within a single sample. If DNA damage occurs according to a random Poisson process at a rate of λ, then there is expected to be an exponential decline in the amount of amplifiable product with increasing product size, and the rate of decline is sharper for higher values of λ (Figure 1). By fitting a model of random degradation to the qPCR data, it should be possible to estimate the frequency of polymerase blocking DNA damage (Figure 2).

In order to provide an initial assessment of our proposed method, we estimate the frequency of DNA damage in DNA extracted from faeces. Faeces contain DNA from a variety of sources, including DNA from the defecating animal, ingested food, parasites and gut microorganisms [21,22]. Of particular interest to zoologists are the DNA from the defecating animal, which can be used as a non-invasive source of DNA from wild species [1,23], and DNA from ingested food, which can be used to study diet [24]. DNA from animal food sources are expected to be highly degraded since these tissues are usually fully digested after passing through the complete digestive system. In comparison, DNA from the defecating animal should be slightly less degraded because this component largely originates from cells shed along the lower digestive tract. We examine DNA extracted from 10 faecal samples collected from captive Steller sea lions (*Eumetopias jubatus*) that had been fed Pacific herring (*Clupea pallasii*). In each sample the amount of sea lion and herring DNA is quantified using species-specific primer sets that amplify fragments of five different lengths. We evaluate if a model of random degradation fits the data and then estimate the frequency of damage in the predator and prey DNA components (see Figure 2 and Methods section).

## Results and Discussion

As expected with degraded DNA template, the amount of amplifiable DNA was inversely related to PCR product size for all targets amplified from the faecal DNA extracts (Figure 2c; Table 1). Expressed as copy number per milligram of extracted faecal matter, the samples contained on average 123209 copies (range 19398 – 281880) of the 61 bp sea lion fragment compared to only 8917 copies (range 692 – 26676) of the 327 bp sea lion fragment. In comparison, the samples contained on average 15109 copies (range 418 – 35498) of the 69 bp herring fragment and just 173 copies (range 38 – 395) of the 304 bp herring fragment. Thus, on average, the faecal extracts contained eight times more sea lion DNA than herring DNA at the smallest fragment sizes and 52 times more at the largest fragment sizes. The large inter-sample range in the amount of predator and prey DNA obtained from different faecal samples is consistent with the amount of variation found in another study that quantified predator DNA in faeces [1]. There was no clear relationship between the amount of sea lion DNA and herring DNA purified from individual samples (Table 1).

**Table 1:** Estimated copy numbers of template in each PCR amplification and results from the random degradation model fits.

**(a) **Sea lion DNA									
Sample	Mean copy number at various amplicon sizes					Model parameters			
	**61 bp**	**91 bp**	**163 bp**	**230 bp**	**327 bp**	**λ**^a^	**CV(λ)**	**1/λ**^b^	**R**^2^
		
1	44727	22461	11965	3746	1387	0.0129	10.3	78	0.92
2	24347	15968	5393	1487	525	0.0148	6.9	67	0.96
3	236825	193410	121534	44745	25936	0.0088	9.1	113	0.94
4	35346	30846	20383	9914	5390	0.0074	10.9	135	0.91
5	43789	26107	11972	4232	1276	0.0135	7.6	74	0.96
6	179118	126256	57217	26322	8684	0.0115	7.9	87	0.95
7	167038	123458	62726	17076	7944	0.0121	7.0	83	0.96
8	29683	25006	14941	9007	2818	0.0089	8.7	113	0.94
9	198820	222720	146709	53608	19759	0.0093	11.0	108	0.91
10	58503	46534	34979	25738	9624	0.0066	11.7	153	0.90

Mean	101820	83277	48782	19588	8334	0.0106	9.1	101	0.94

**(b) **Herring DNA									

Sample	Mean copy number at various amplicon sizes					Model parameters			
	**69 bp**	**123 bp**	**184 bp**	**226 bp**	**304 bp**	**λ**^a^	**CV(λ)**	**1/λ**^b^	**R**^2^
		
1	8157	522	143	93	42	0.0221	15.9	45	0.83
2	9927	994	257	240	120	0.0192	19.7	52	0.76
3	14005	2016	687	381	178	0.0179	11.2	56	0.91
4	1575	418	246	182	69	0.0123	10.9	81	0.91
5	11649	952	243	149	46	0.0222	12.5	45	0.89
6	11754	1700	605	307	120	0.0188	9.7	53	0.93
7	18588	4016	1272	1165	234	0.0180	11.7	56	0.90
8	25203	8883	2306	1366	429	0.0173	6.0	58	0.97
9	26295	5497	1299	655	266	0.0197	9.2	51	0.94
10	711	432	238	138	107	0.0086	11.9	117	0.90

Mean	12786	2543	729	468	161	0.0176	11.9	61	0.89

^a^λ is an estimate of the probability of a nucleotide being damaged.

^b ^1/λ is an estimate of the average undamaged fragment size (in base pairs)

**Figure 3:**
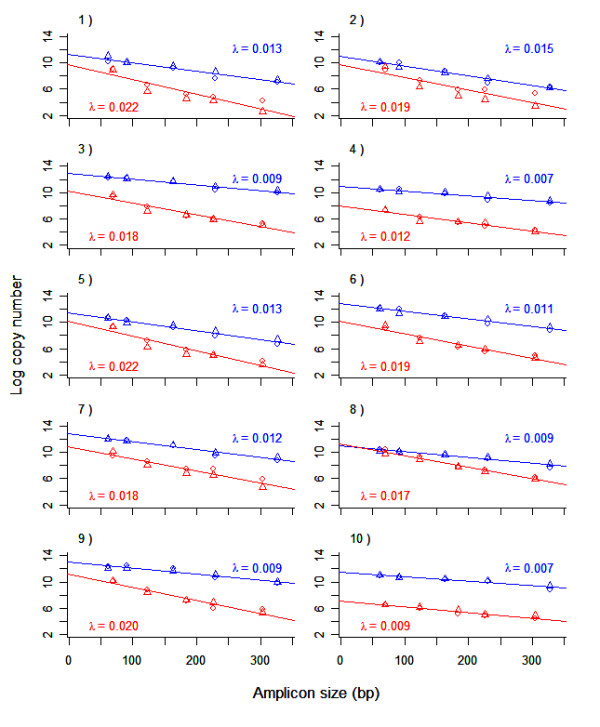
**Quantitative PCR results and estimates of DNA damage in faecal DNA**. Shown is the number of amplifiable copies (logarithmic scale) verus amplicon size for sea lion DNA (blue) and herring DNA (red) extracted from ten sea lion faecal samples. The estimated probability of a nucleotide being damaged (λ) is also shown for each target species in each sample.

Results from fitting the random degradation model (see Methods section) to the data for each sample and target species indicate that the model describes the data well, with R^2 ^values generally above 0.90 (Figure 3; Table 1). The process of DNA degradation will be sample specific, but within any sample, damage that prevents PCR amplification will be caused by a large variety of mechanisms. Therefore, we expect that degradation will essentially be random in a wide variety of highly degraded DNA samples. In the faecal samples, the estimated probability of a nucleotide being damaged (λ) varied between samples for a given target species (0.0066 to 0.0148 for sea lion DNA; 0.0086 to 0.0222 for herring DNA). Within a sample, the λ estimate for herring was always greater than that for sea lion (Figure 3). On average, the frequency of damage was 1.7 times greater for the herring DNA compared with the sea lion DNA from the same sample; a paired t-test indicates the difference in λ values is significant (t = 8.4 with 9 df, p < 0.001). The mean fragment size in each sample can be estimated by 1/λ (Table 1). Averaging over all samples, the mean fragment size for the herring DNA is 61 bp versus 101 bp for the sea lion DNA.

There is no obvious relationship between amount of DNA (log(N)) and level of degradation (λ). Correlation between log(N) and λ for sea lion is -0.06; for herring it is 0.76 but this is being driven by two samples (4 and 10) with very low amounts of herring DNA. Leaving these two points out gives a correlation of -0.53.

**Figure 4:**
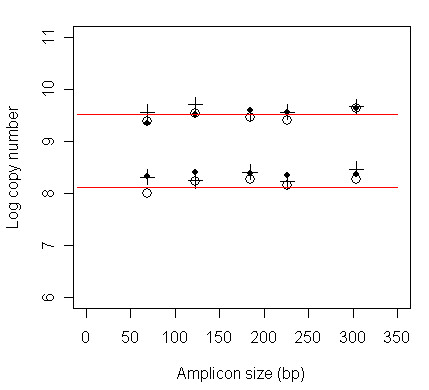
**Quantitative estimates of the amount of amplifiable herring DNA in three spiked faecal DNA extractions**. Horizontal lines show the actual amount of herring DNA added as template (either 3380 or 13520 copies of the plasmid control). Symbols represent the corresponding estimates of the amount of herring DNA in three samples measured with assays targeting PCR products of five different sizes (69 bp, 123 bp, 184 bp, 226 bp and 304 bp).

A decrease in PCR signal with an increase in the length of the product could result from selective inhibition of longer PCR amplifications caused by the coextraction of inhibitory chemicals rather than the absence of undamaged DNA in samples [e.g. [25]]. To determine if extract induced PCR inhibition could have affected the results of the current study we spiked known amounts of herring DNA into faecal extracts containing no endogenous herring DNA. We found no evidence of inhibitory effects caused by chemicals in the three sea lion faecal DNA extracts that we tested (Figure 4).

While it is well known that short fragments are present in larger amounts than long fragments in degraded DNA samples, the formalization of this relationship clarifies the relative nature of quantitative measurements obtained when analysing degraded templates using qPCR (i.e. the estimated amount of DNA will vary with marker size in a sample-specific fashion). This means that comparisons of DNA quantity (within and between samples) are dependent on the size of the fragments targeted by qPCR. This can have practical implications – for example, a previous study [1] used qPCR targeting an 81 bp nuclear gene fragment in order to determine the amount of chimpanzee nuclear DNA present in faeces collected from wild chimpanzees. When the measured amount of DNA was low, the quantity of 81 bp DNA was not a good indicator of the ability to recover chimpanzee microsatellite markers which were 101–266 bp in size. This indicates the level of DNA degradation differed between samples, and that quantitative pre-screening of non-invasive DNA extracts should target fragments at least as large as the markers to be used in the final screening.

Our quantitative estimates show that there is less prey DNA compared to predator DNA in sea lion faeces for all PCR fragment sizes tested. Previous studies [23] have found the low quantity of predator DNA in faeces problematic, which suggests that the even more limited amount of prey DNA may be a serious difficulty for DNA-based diet studies relying on faecal samples. Fortunately, multi-copy nuclear or mitochondrial genes are usually appropriate markers for diet studies, as opposed to the single-copy markers which are often targeted for studies on the predator. This advantage may allow for reliable recovery of prey-specific DNA sequences from faecal samples. In DNA-based diet studies, the appropriate size of a PCR target is a trade off between the amount of information obtained from the DNA (usually directly related to fragment size) and the quantity of template DNA available (inversely related to fragment size). The model we have presented can be used to predict the approximate amount of DNA present for a given fragment size (based on an appropriate λ value and at least one quantitative PCR measurement from a sample). This will allow for an objective appraisal of optimal PCR target size for samples of differing quality.

**Figure 5:**
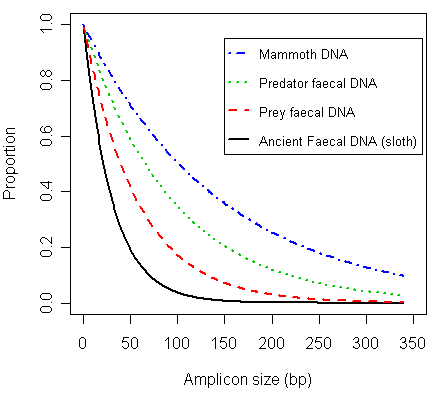
**Plots showing estimated proportion of amplifiable fragments versus amplicon size for various DNA extracts**. The predator and prey faecal DNA curves use the mean λ values determined in the current study for sea lion DNA (λ = 0.0106) and herring DNA (λ = 0.0176). The ancient faecal DNA λ was estimated from published data [17] on the quantity of sloth mtDNA in a late Pleistocene sloth coprolite (λ = 0.033). The mammoth DNA λ (0.007) was also estimated from published data [26].

In ancient DNA work it has been recognised that an assessment of both the amount of DNA present and the amount of damage in a sample is useful in order to define the limits of subsequent analyses and the authenticity of the sample [7,9]. There are two ancient DNA study we are aware of which have quantified the number of fragments of different lengths present in samples [17,26]. The first study analysed three different fragments (114, 252 and 522 bp) of sloth mtDNA from a late Pleistocene sloth coprolite [17]. The largest fragment size examined contained on average only 0.5 ± 0.5 copies of DNA. With almost zero copies and such a large relative error, this point is not informative for quantification of damage; however, based on the other two points we can estimate the probability of a nucleotide being damaged (λ) to be 0.033, meaning an average fragment size of about 30 bp. Although this estimate is likely inaccurate due to the limited data, it is consistent with our *a priori *expectation that ancient faecal DNA would be considerably more degraded than modern faecal DNA (Figure 5). In the second study, DNA recovered from a well preserved mammoth bone was examined [26] and mtDNA molecules were quantified for six different fragments sizes (84, 151, 297, 490, 677 and 921 bp). The resulting data are highly consistent with the random degradation model (R^2 ^= 0.99, λ = 0.007). The λ value is indicative of ancient DNA in remarkably good condition, with an average fragment size of 150 bp (Figure 5). The data from these studies demonstrate that the approach we have outlined is useful for determining DNA damage in molecules from ancient sources and will allow for meaningful comparison of DNA damage in samples from different studies.

Another potential application of our methodology is in studies on the process of DNA decay. While DNA damage should correlate with age of template, the connection is often somewhat unclear [9,14,27,28]. A possible reason in some studies is that quantity is being used as a proxy for quality [12,15]. The problem with doing so is that the high variance in the amount of DNA between different samples can obscure the decrease in the amount of DNA over time. Our results showed a roughly 10-fold variance in amount of DNA between samples, whereas the variance in λ values was only 2-fold. This suggests that DNA decay might be better studied by determining DNA degradation in samples of different ages rather than focusing on the amount of DNA present. Several studies on DNA decay have used various biochemical assays to measure DNA degradation [7-9]. While these studies provide valuable information on the chemical process of DNA decay, the methods they employ are often not easily accessible. Our technique should be more accessible and could be modified to allow for the quantification of various forms of DNA damage. For example, the frequency of cytosine deamination could be quantified through comparison of the original sample with aliquots treated with uracil N-glycosylase [29]. Other forms of damage could also be measured using other lesion-specific endonucleases (or chemical equivalents) or lesion-specific repair enzymes [7].

## Conclusion

In this article we presented a PCR-based approach for quantitative measurement of gene-specific DNA damage in highly degraded, mixed template samples. The method was used to estimate the amount of DNA damage in two components of DNA extracted from sea lion faeces: prey DNA (expected to be highly degraded) and predator DNA (expected to be slightly less degraded). The distribution of fragment lengths in these faecal DNA templates fit well with our assumption of a random degradation process, and differences in the estimated frequency of predator versus prey DNA damage within samples were congruent with expectations. The data highlight the rapid decrease in copy number as fragment size increases in these samples, and show that predator DNA is more prevalent than prey DNA in sea lion faeces. Based on this initial assessment, we envisage that the general methodology could be applied to study a variety of degraded DNA templates. This will allow researchers to evaluate alternate sources of DNA, different methods of sample preservation and different DNA extraction protocols. The technique should also be more accessible than alternate biochemical methods for studying the process of DNA decay.

## Methods

### DNA Samples

The sea lion faecal samples are a subset of those from a previous study [30]. Ten samples were analysed for endogenous DNA from sea lion and Pacific herring. These samples were collected from captive sea lions being fed a diet consisting of 47% herring by mass for a period of at least 48 h before collection and were previously shown to contain herring DNA [30]. Three additional sea lion faecal samples were collected from animals being fed a diet of 100% walleye pollock (*Theragra chalcogramma*). These samples were used in spiking experiments for the analysis of length inhibition (see section below). Sample storage and DNA extraction for the sea lion samples has been described previously [30].

### Primer design

PCR primers which amplify fragments from the 3' region of the mitochondrial 16S gene (large subunit rDNA gene) were designed using the software Primer3 [31]. For each target, a common forward primer was selected for use with five reverse primers, producing products in the range of 61–357 bp (Figure 2a). Primer sequences and product sizes are given in Table 2. The primers were designed with reference to aligned sequences from the sea lion and all fish species in the sea lions' diet. The forward primers were specific to the target species (i.e. they bind to regions conserved within but not between species) and the specificity of the primer sets was tested empirically against non-target DNA. The herring primer sets were tested against three faecal extracts from sea lions fed only pollock, and the sea lion primers against herring genomic DNA. None of the primers amplified products from the non-target templates tested, and melting curve analysis performed on products obtained during qPCR indicated that each primer set produced a single product.

**Table 2:** Sequences of primers used to quantify DNA degradation.

Target	Forward Primer (5' → 3')	Reverse Primers (5' → 3')	Length of PCR Product (bp)
Sea Lion	CAAGTCAACCAAAACGGGATA	CACCCCAACCTAAATTGCTG	61
		TCACTCGGAGGTTGTTTTGTT	91
		CTTGTTCCGTTGATCAAAGATT	163
		TCGAGGTCGTAAACCCTGTT	230
		GATTGCTCCGGTCTGAACTC	327
Herring	ACCAATCACGAAAAGCAGGT	CGAAGACGTTTGTGCCAGTA	69
		TAGGGTAGCCCCAATCCTCT	123
		GCATGTAGCCGGATCATTTT	184
		GGATTGCGCTGTTATCCCTA	226
		AATAGCGGCTGCACCATTAG	304

In general, when using the outlined methodology to quantify DNA damage in highly degraded samples, it is best to determine copy numbers for small fragments. This is because the copy number will decrease rapidly as fragment size increases and qPCR measurements at low copy numbers (< 100 copies per reaction) are inaccurate due to the larger relative impact of stochastic factors in PCR [32]. Another concern is the potential influence of reconstructive polymerization when the amount of template is low and competition for reaction components is minimal [33].

### Quantification of mtDNA

The quantity of extracted 16S mtDNA was estimated using SYBR^® ^Green based qPCR assays. Amplifications were run using the Chromo4™ detection system (MJ Research). The PCR mix (20 μL) consisted of 10 μL QuantiTect^® ^SYBR^® ^Green PCR mix (Qiagen), 0.5 μM of each primer, 1 × BSA (New England Biolabs) and 4 μL template DNA (diluted 1:5). Thermal cycling conditions were: 94°C for 15 min followed by 35 cycles of: 94°C, 30 s/55°C, 30 s/72°C, 45 s; optical data was acquired following each 72°C extension step (Figure 2b). A subset of samples was separated on 1.8% agarose gels to confirm products were of the expected size and to ensure no primer dimers were present.

A plasmid standard encompassing the relevant 16S mtDNA region was generated from genomic DNA for each target species. This was accomplished by amplifying the region using the conserved primers (16S1F GGACGAGAAGACCCT and 16Sbr-3' CCGGTCTGAACTCAGATCACGT) and cloning the PCR products using the TOPO TA cloning kit (Invitrogen). Plasmid DNA was isolated by alkaline lysis and the concentration of plasmid DNA was determined by fluorescence of PicoGreen (Molecular Probes) in a PicoFluor fluorometer (Turner Designs). Standard curves were generated using a 5-fold dilution series of plasmid encompassing the concentration range of the faecal template. Separate standard curves were constructed for each of the different sized PCR amplifications and for each target species. Independent curves were calculated during each PCR run. For each assay there was a linear relationship between the log of the plasmid DNA copy number and the C_t _value over the concentration range of the standards (mean R^2 ^= 0.994). The binding site for the 327 bp sea lion reverse primer was incomplete in the plasmid control, so quantification of this DNA fragment was based on the standard curve generated for the 230 bp sea lion fragment.

For individual extractions, the complete set of fragment sizes for a particular target was quantified in a single run (using a PCR reagent mix differing only in primer composition). This minimized the variation in reaction conditions between the different sized fragments that were being compared. Two independent runs were carried out for each assay. For quantitation, the threshold cycle (C_t_) was set at ten standard deviations above the mean fluorescence over cycle range 1–10. To avoid contamination with undamaged DNA, faecal DNA template was added to tubes first and their caps were sealed before plasmid DNA was added to appropriate standard tubes in a separate room. Aerosol-resistant pipette tips were used with all PCR solutions, and template free negative control reactions were included for each unique PCR mix. None of our negative control samples produced fluorescence signals that reached the threshold detection level in 35 cycles.

### Model for quantitative estimates of DNA damage

DNA damage resulting in strand breaks or chemical modifications that would prevent PCR amplification can be caused by a number of mechanisms. We assume that in highly degraded samples such DNA damage occurs according to a random Poisson process at a rate of λ per nucleotide (i.e. λ is the probability of a nucleotide being damaged). The resulting distribution of undamaged fragment sizes (x) is defined by an exponential distribution with parameter λ:

f(x) = λe^-λx ^    **1**

This model has been used to characterise DNA damage induced by some mutagenic agents [10,19], and a very similar model has been used to describe random fragmentation resulting from DNase I digestion [34]. It follows from the properties of an exponential distribution that the average undamaged fragment size is 1/λ, and that the variance of undamaged fragment sizes is 1/λ^2^.

Since PCR will amplify any DNA which is undamaged in a region equal to or greater in size than the target region, we are interested in the probability of a fragment of size × or greater being present. This is given by e^-λx^, the complement of the cumulative exponential distribution. In a PCR designed to amplify a target region of size × (i.e. amplicon size × bp), the expected proportion of amplifiable copies is e^-λx^. Thus, as product size increases, there is an exponential decline in the proportion of amplifiable product and the rate of decline is determined by the value of λ (Figure 1). If the total number of DNA copies present in the sample is N, then the expected number of amplifiable copies, denoted by A_x_, is Ne^-λx^. Using a logarithmic transformation this relationship can be expressed in linear form as:

log(A_x_) = log(N) – λx     **2**

The observed value of A_x _will vary due to the random nature of the degradation process. If the process is truly Poisson, then the amount of variance can be calculated theoretically – in theory, A_x _is binomially distributed with sample size N and 'success' probability e^-λx^, so the variance is Ne^-λx^(1-e^-λx^). However, the variability observed in practice is expected to be greater because the degradation process is not likely to follow a Poisson process exactly and, even if it did, there will be experimental measurement error. Here we assume that the error in log(A_x_) is normally distributed with mean 0 and variance σ^2^; this is consistent with previous studies [10]. Assuming this error structure, equation **2 **can be fit using simple least-squares regression (Figure 2d).

For each of the ten sea lion faecal samples, we obtained two estimates of copy number (A_x_) corresponding to five fragment sizes (x) for both sea lion (predator) DNA and herring (prey) DNA. We fit the model given in equation **2 **to the data from each sample and target species to obtain estimates of log(N) and λ, with λ being the parameter of key interest. Coefficients of variation for the parameter estimates and R^2 ^values were also obtained for each of the model fits.

### Analysis of length-inhibition

To investigate the potential inhibitory effects of the faecal DNA extracts on PCR we carried out spiking experiments. This involved adding known amounts of undegraded herring DNA (3380 or 13520 copies of the plasmid control) to sea lion faecal DNA extracts that contained no endogenous herring DNA (n = 3). The amount of recoverable herring DNA of the five sizes was estimated as outlined above.

## Competing interests

The author(s) declare that they have no competing interest.

## Authors' contributions

BD designed the study, carried out the lab work and drafted the manuscript. PE carried out the major part of the data analysis. All authors participated in the development of concepts presented in the paper and contributed significantly to the writing of the final version of the manuscript. All authors read and approved the final manuscript.

## References

[B1] Morin PA, Chambers KE, Boesch C, Vigilant L (2001). Quantitative polymerase chain reaction analysis of DNA from noninvasive samples for accurate microsatellite genotyping of wild chimpanzees (Pan troglodytes verus). Mol Ecol.

[B2] Jarman SN, Gales NJ, Tierney M, Gill PC, Elliott NG (2002). A DNA-based method for identification of krill species and its application to analysing the diet of marine vertebrate predators. Mol Ecol.

[B3] Withler RE, Candy JR, Beacham TD, Miller KM (2004). Forensic DNA analysis of Pacific salmonid samples for species and stock identification. Environ Biol Fishes.

[B4] Glenn TC, Stephan W, Braun MJ (1999). Effects of a population bottleneck on Whooping Crane mitochondrial DNA variation. Conserv Biol.

[B5] Shapiro B, Drummond AJ, Rambaut A, Wilson MC, Matheus PE, Sher AV, Pybus OG, Gilbert MTP, Barnes I, Binladen J, Willerslev E, Hansen AJ, Baryshnikov GF, Burns JA, Davydov S, Driver JC, Froese DG, Harington CR, Keddie G, Kosintsev P, Kunz ML, Martin LD, Stephenson RO, Storer J, Tedford R, Zimov S, Cooper A (2004). Rise and fall of the Beringian steppe bison. Science.

[B6] Lindahl T (1993). Instability and Decay of the Primary Structure of DNA. Nature.

[B7] Pääbo S (1989). Ancient DNA - Extraction, Characterization, Molecular-Cloning, and Enzymatic Amplification. Proc Natl Acad Sci USA.

[B8] Höss M, Jaruga P, Zastawny TH, Dizdaroglu M, Pääbo S (1996). DNA damage and DNA sequence retrieval from ancient tissues. Nucleic Acids Res.

[B9] Mitchell D, Willerslev E, Hansen A (2005). Damage and repair of ancient DNA. Mutat Res-Fundam Mol Mech Mutagen.

[B10] Ayala-Torres S, Chen YM, Svoboda T, Rosenblatt J, Van Houten B (2000). Analysis of gene-specific DNA damage and repair using quantitative polymerase chain reaction. Methods.

[B11] Govan HL, Vallesayoub Y, Braun J (1990). Fine-Mapping of DNA Damage and Repair in Specific Genomic Segments. Nucleic Acids Res.

[B12] Marota I, Basile C, Ubaldi M, Rollo F (2002). DNA decay rate in papyri and human remains from Egyptian archaeological sites. Am J Phys Anthropol.

[B13] Hoogendoorn M, Heimpel GE (2001). PCR-based gut content analysis of insect predators: using ribosomal ITS-1 fragments from prey to estimate predation frequency. Mol Ecol.

[B14] Gilbert MTP, Hansen AJ, Willerslev E, Rudbeck L, Barnes I, Lynnerup N, Cooper A (2003). Characterization of genetic miscoding lesions caused by postmortem damage. Am J Hum Genet.

[B15] Wandeler P, Smith S, Morin PA, Pettifor RA, Funk SM (2003). Patterns of nuclear DNA degeneration over time - a case study in historic teeth samples. Mol Ecol.

[B16] Pääbo S, Innis MA, Gelfand DH, Sninsky JJ and White TJ (1990). Amplifying ancient DNA. PCR-Protocols and Applications - A Laboratory Manual.

[B17] Poinar H, Kuch M, McDonald G, Martin P, Paabo S (2003). Nuclear gene sequences from a late Pleistocene sloth coprolite. Curr Biol.

[B18] Jennerwein MM, Eastman A (1991). A Polymerase Chain Reaction-Based Method to Detect Cisplatin Adducts in Specific Genes. Nucleic Acids Res.

[B19] Fernando LP, Kurian PJ, Fidan M, Fernandes DJ (2002). Quantitation of gene-specific DNA damage by competitive PCR. Anal Biochem.

[B20] Mambo E, Gao XQ, Cohen Y, Guo ZM, Talalay P, Sidransky D (2003). Electrophile and oxidant damage of mitochondrial DNA leading to rapid evolution of homoplasmic mutations. Proc Natl Acad Sci USA.

[B21] Poinar HN, Kuch M, Sobolik KD, Barnes I, Stankiewicz AB, Kuder T, Spaulding WG, Bryant VM, Cooper A, Paabo S (2001). A molecular analysis of dietary diversity for three archaic Native Americans. Proc Natl Acad Sci USA.

[B22] Jarman SN, Deagle BE, Gales NJ (2004). Group-specific polymerase chain reaction for DNA-based analysis of species diversity and identity in dietary samples. Mol Ecol.

[B23] Taberlet P, Griffin S, Goossens B, Questiau S, Manceau V, Escaravage N, Waits LP, Bouvet J (1996). Reliable genotyping of samples with very low DNA quantities using PCR. Nucleic Acids Res.

[B24] Symondson WOC (2002). Molecular identification of prey in predator diets. Mol Ecol.

[B25] Pusch CM, Bachmann L (2004). Spiking of contemporary human template DNA with ancient DNA extracts induces mutations under PCR and generates nonauthentic mitochondrial sequences. Mol Biol Evol.

[B26] Poinar HN, Schwarz C, Qi J, Shapiro B, MacPhee RDE, Buigues B, Tikhonov A, Huson DH, Tomsho LP, Auch A, Rampp M, Miller W, Schuster SC (2006). Metagenomics to paleogenomics: Large-scale sequencing of mammoth DNA. Science.

[B27] Gilbert MTP, Janaway RC, Tobin DJ, Cooper A, Wilson AS (2006). Histological correlates of postmortem mitochondrial DNA damage in degraded hair. Forensic Sci Int.

[B28] Pääbo S, Higuchi RG, Wilson AC (1989). Ancient DNA and the Polymerase Chain-Reaction - the Emerging Field of Molecular Archaeology. J Biol Chem.

[B29] Hofreiter M, Jaenicke V, Serre D, von Haeseler A, Paabo S (2001). DNA sequences from multiple amplifications reveal artifacts induced by cytosine deamination in ancient DNA. Nucleic Acids Res.

[B30] Deagle BE, Tollit DJ, Jarman SN, Hindell MA, Trites AW, Gales NJ (2005). Molecular scatology as a tool to study diet: analysis of prey DNA in scats from captive Steller sea lions. Mol Ecol.

[B31] Rozen S, Skaletsky HJ, Krawetz S and Misener S (2000). Primer3 on the WWW for general users and for biologist programmers.. Bioinformatics Methods and Protocols: Methods in Molecular Biology.

[B32] Peccoud J, Jacob C (1996). Theoretical uncertainty of measurements using quantitative polymerase chain reaction. Biophys J.

[B33] Golenberg EM, Bickel A, Weihs P (1996). Effect of highly fragmented DNA on PCR. Nucleic Acids Res.

[B34] Moore GL, Maranas CD (2000). Modeling DNA mutation and recombination for directed evolution experiments. J Theor Biol.

